# Evaluating Origin–Destination Matrices Obtained from CDR Data

**DOI:** 10.3390/s19204470

**Published:** 2019-10-15

**Authors:** Marco Mamei, Nicola Bicocchi, Marco Lippi, Stefano Mariani, Franco Zambonelli

**Affiliations:** 1 Dipartimento di Scienze e Metodi dell’Ingegneria, University of Modena and Reggio Emilia, 42122 Reggio Emilia, Italy; marco.lippi@unimore.it (M.L.); stefano.mariani@unimore.it (S.M.); franco.zambonelli@unimore.it (F.Z.); 2 Centro En&Tech, University of Modena and Reggio Emilia, 42124 Reggio Emilia, Italy; 3 Centro Softech-ICT, University of Modena and Reggio Emilia, 41125 Modena, Italy; nicola.bicocchi@unimore.it; 4 Dipartimento di Ingegneria Enzo Ferrari, University of Modena and Reggio Emilia, 41125 Modena, Italy

**Keywords:** mobility patterns, CDR data, OD matrices

## Abstract

Understanding and correctly modeling urban mobility is a crucial issue for the development of smart cities. The estimation of individual trips from mobile phone positioning data (i.e., call detail records (CDR)) can naturally support urban and transport studies as well as marketing applications. Individual trips are often aggregated in an origin–destination (OD) matrix counting the number of trips from a given origin to a given destination. In the literature dealing with CDR data there are two main approaches to extract OD matrices from such data: (a) in time-based matrices, the analysis focuses on estimating mobility directly from a sequence of CDRs; (b) in routine-based matrices (OD by purpose) the analysis focuses on routine kind of movements, like home-work commute, derived from a trip generation model. In both cases, the OD matrix measured by CDR counts is scaled to match the actual number of people moving in the area, and projected to the road network to estimate actual flows on the streets. In this paper, we describe prototypical approaches to estimate OD matrices, describe an actual implementation, and present a number of experiments to evaluate the results from multiple perspectives.

## 1. Introduction

In recent years, there has been a growing interest in the development of ICT technologies that can succeed in collecting, processing, and analyzing mobility data with simple, efficient, and privacy-preserving procedures.

The widespread diffusion of mobile phones and cell networks provides a practical way to collect location-based information from large user populations. The analysis of such data is a key asset in the development of several applications, including location-based services, traffic forecasting, urban planning and management [1,2,3,4]. In particular, the estimation of individual trips from mobile phone positioning data (i.e., call detail records (CDR)) is an important application in this area, and it can naturally support urban and transport studies, as well as marketing applications, by allowing us to estimate the passage of potential customers on a given path [5,6,7,8,9]. CDRs are information automatically collected by the mobile phone network about where and when a cell phone connects to the network. Therefore, CDRs provide approximate location samples of the phone’s owner, and a sequence of CDRs can provide their mobility pathways and trips.

The estimation of the demand for mobility and transportation has been analyzed in a large number of works since it represents a critical task for transportation systems and urban planning. To address this problem, several methods have been proposed in the literature. These models combine accurate statistical sampling methods and travel surveys to infer trip information between areas of the city [10,11,12,13,14,15,16,17,18]. While the surveys providing data for these models offer detailed travel logs for representative population samples, they are expensive to administer and participate in. Moreover, most of the approaches start from a trip generation model (e.g., [19]) that can only approximate the actual mobility patterns.

More recently, there has been a growing interest in big data sources that capture the movement of vehicles and people in near real-time, and promise more cost-effective solutions to estimate mobility demand [5,6,7,8,9,20,21,22,23,24,25,26]. On the one hand, these approaches can sidestep the trip-generation-modeling phase and estimate mobility directly from observed data (e.g., CDR). On the other hand, they can also be used to refine and tailor trip generation models to a specific setting (e.g., by taking into account where people live and work as measured by CDRs). This kind of approach is very promising, but a lot of work is still required to better evaluate resulting mobility estimation. In particular, the work in [26] discusses a number of limitations for this kind of estimation based on big data, and it raises the attention on the need for more experiments to validate and assess the validity of such data-driven traffic estimation. Our work aims to provide an overview of the different methods to estimate mobility demand in this growing research area, and it applies a number of mechanisms to validate the results under multiple perspectives.

In this work we focus on the estimation of origin-destination (OD) matrices: individual trips are often aggregated in an origin–destination matrix counting the number of trips from a given origin to a given destination. An OD-matrix assumes that the region under study is partitioned into a finite set of non-overlapping zones $S_{i}:i=1,..,n$ and records the number of trips from any origin zone $S_{i}$ to any destination zone $S_{j}$ that takes place in a given day and time interval. So, for example, in the OD matrix associated with a given day from 8 a.m. to 9 a.m., $OD_{ij}$ counts the number of trips from $S_{i}$ to $S_{j}$ during that time interval.

There are basically two kinds of approaches to extract origin-destination trips and aggregate them in OD matrices:

- Time-based matrices (tOD) focus on a given time window in a specific day. They estimate the motion of users directly from observed CDRs generated within that time window [5,6,7]. The main advantage of these approaches is that they can be computed in real-time and capture the specific trips actually taking place at that time. The main disadvantage is that they capture only a fraction of the population (people not using the phone in the time frame are invisible—and it is not easy to scale up the estimates).
- Routine-based matrices (rOD, or OD by purpose) focus on routine movements like home-work commute [8]. They are computed from a trip-generation model estimating routine movements for each person in the area on a given day at a given time. On this basis, they are computed by aggregating all the routine movements that are assumed to take place at that time. The main advantage of these models is that they involve the whole telecom operator market share and it is relatively easy to scale up the estimates to the whole population. The main disadvantage is that they represent the “modeled” flow for that routine, and thus they cannot easily cope with the peculiarities of a given day.

The goal of this work is to present in a coherent framework the main approaches to compute time-based matrices (tOD) and routine-based matrices from CDR data, in particular focusing on the home–work commute (hwOD). In particular, we exploit a specific implementation that is suitable to the data at our disposal, and evaluate the results of OD matrices estimation across different experiments.

Specifically, Section 2 presents an extensive analysis of the state of the art in OD estimation from CDR data. in Section 3, we present the proposed algorithms. In Section 4 we describe experimental results and compare them with census-based data. Section 5 concludes the paper and highlights paths for future research.

## 2. Related Work

In this section we describe a number of works that aim to extract OD matrices (or equivalent information) from CDR data. Different works use different kinds of CDR data: CDRs at sector/cell level; CDRs + mobility management (MM) procedure messages (i.e., IMSI attach/detach and location update) [27]; CDRs + DDRs (DDR are data detail records, recording data traffic rather than just calls and sms); triangulated CDR data with higher spatial resolution (200 m) [6], handoff CDR data (data about cell sectors relaying extended calls). Almost all the works follow a procedure similar to the one described: (i) OD estimation; (ii) scaling; (iii) road traffic assignment; (iv) correlation analysis with Census data or other data sources (traffic sensors, traffic cameras, etc.)—typically those used in scaling. Some analysis are conducted at the city level, i.e., monitoring traffic from one city (county) to another. More refined approaches are conducted at the district (tract) level, measuring traffic within a city. Related works organized according to the above steps are represented in Table 1.

In [5,6], authors compute tOD matrices on the basis of CDR+DDR data and apply clustering techniques to filter out noise due to localization. Then, they aggregate movements in time to detect OD matrices and scale results on the basis of Census information recording the origin-destination matrix of journeys for work reasons (http://www.fhwa.dot.gov/ctpp). Results at the city-city level (number of trips from one city to the other) indicate a correlation with census data associated with r=0.87, while correlation at the district-district level to r=0.6.

In the work described in [7,8] authors derive OD matrices from CDR data and the corresponding traffic assignment to the road network. More in detail, similarly to [5], in [7] OD matrices over a specific time-interval (tOD) are computed. Specifically, the area under analysis is rasterized, and CDRs are projected into the associated grid cells. Trips between two towers are recorded as movements between the two associated grid cells. Grid cells are aggregated on the basis of the city area, and the tOD matrix is computed on this basis. Numbers in the tOD matrix are scaled according to Census data taking into account the number of people living in a certain area and the vehicle usage rate of individuals living in that area (https://ctpp.transportation.org). The result is a transition probability matrix (summing up to 1) that is finally scaled by the number of trips typically generated in the city in that time interval to obtain the final tOD. As reported, this approach tends to be biased towards short trips (as the user might generate a CDR while in transit rather than at start–stop locations). Results indicate a correlation with r=0.6 with census data.

In [8] OD matrices for different movement categories are computed (e.g., home-work commute). First, relevant places for each user and typical travel times are computed. Then, OD matrices are computed in a similar way as before from and to the identified locations. The number of trips in the matrices reflects the number of users being monitored. Therefore, matrices are scaled to the total population by considering the National Household Travel Survey (NHTS) in order to match the overall population reported in the survey. Results at the city-city level indicate correlation with census data associated to r=0.98, while correlation at the district-district level with r=0.55.

In [25], authors survey a number of works extracting OD matrices from different data sources. Then, they propose an approach to extract tOD matrices from CDRs in a city. OD matrix identification is similar to [7], however, the approach to scale OD values is different. In this work, they capture traffic at some given locations using cameras. Then, they run traffic (micro-)simulations on the basis of the OD matrix to estimate traffic in the city (simulation performs incremental road assignment). Finally, they optimize the scaling factor so that simulation results match the camera counts. The approach is validated by matching traffic with other camera counts not used in calibration. Results are: root mean square error = 335.09, root mean square percent error = 13.59%.

In [34], authors extract OD matrices from GPS taxi traces in New York city. They apply a maximum-entropy approach and, similarly to [7,8], they obtain a transition probability matrix by combining maximum-likelihood analysis on data and a fitted gravity model. Their goal is to infer how a large dataset can be reconstructed from a smaller dataset, so they do not compare with Census data nor they scale the results with Census information. They compare results from a (small) sub-sample of the data with the original dataset. Results at a fine-grained scale (road intersection) indicate that a sample of 25% of the data correlated with the original data with r=0.91.

In [28], OD matrices for the home–work commute are computed. First, home and workplaces are identified for each user. Then, a simple free-flow road assignment is computed. Similarly, in [29], OD matrices for home-work commute are computed. Special care has been taken to consider only those users having enough data for which the estimation of the home–work commute pattern was statistically significant. A similar approach is taken in [30], in which different cities are compared from a commuting efficiency point of view.

In [31], authors develop a mechanism do identify stop points and movements of users, and to carefully assign movements to road segments. So, basically, they address all the steps of OD matrices construction apart from scaling issues. Basically, the key aspect of their work is to adapt the road graph (processed by A*) so that road weights take into account the cells visited in the path (the weight to roads overlapping with cell sectors where the user generates an event drops). Another aspect is to rely on the ground-truth GPS traces to evaluate the approach. Overall, they obtain a 70 m median error and 75% of errors below 180 m. This kind of approach could be associated with a scaling procedure to obtain a complete OD matrix.

Similarly in [32], authors develop a mechanism for map matching and scaling on the basis of CDR handoff data. Their work is based on a training phase, in which the handoff signature associated with a given road trip is computed and a (nearest neighbor) classifier is trained on that data. Then a testing phase in which a large set of CDR data is classified as belonging to one road. Road usage patterns are validated via correlation with Census data at the road level, producing r = 0.77. Also, in this case, the approach can be extended to derive the OD matrix in the area. Another interesting approach in this direction is presented in [35,36,37]. Authors tackle the map matching problem via a Bayesian network or an Hidden Markov Model (HMM). They try to infer the hidden roads from the CDR observations.

The work described in [33] applies a simple approach to infer tOD matrices from CDR count. Specifically, they compute the tOD by analyzing the cells visited by each user (at a coarse granularity, cells are grouped by district) and filtering out pass-by areas where the user spends little time. What is most interesting in this work is the comparison of such tOD with the gravity model and even more to a gravity model based on mutual calls among regions rather than on people counts. Results indicate r=0.93 correlation between the tOD and the modified gravity model. On the basis of these results, authors conduct an interesting experiment to optimize the road network of the country, finding important validation with actual government policies (i.e., the roads that the model predicts should be created or upgraded are those in the actual government plans). A similar approach has been exploited in [38] where the OD matrix is used to infer how to improve public transportation routes.

The work described in [39] tackles a different problem. They try to estimate train commuters on the basis of CDR data. Their approach is based on finding similarities between CDR trajectories and speed and train lines. Results indicate that they are able to detect train commuters with 75% accuracy. This approach could enrich the OD matrix estimation by better identifying transport mode.

Despite these recent efforts, further research is needed to better evaluate the performance of OD matrices derived from these data sources [26].

## 3. Methodology

In this section we first describe the CDR data at the basis of our work. We then present a specific implementation of the techniques to obtain time-based and routine-based OD matrices. Finally, we describe an algorithm to map OD trips to the road network.

### 3.1. CDR Data

We obtained a large set of mobility data from an Italian telecom operator. In particular, we analyzed data from three regions of Italy (Piemonte, Lombardia and Emilia Romagna inhabited by about 20 million people), spanning several months. Mobility data is obtained from CDRs and MM procedure messages. CDRs are routinely collected by cellular network providers for billing purposes. A CDR is generated every time a phone initiates or receives a voice call or a text message. The IMSI attach/detach procedure marks the phone as attached/detached to the network on power up/power down of the phone or SIM inserted/removed. Location updates are messages exchanged for keeping the network informed of where the phone is roaming. CDR and MM messages are read on network interfaces through specific probes and they also contain the identity of the phone, the identity of the cell through which the phone is communicating and the related timestamp. As MM messages contain the same information as CDRs, for simplicity of writing we will refer to them as CDRs as well.

**Figure 1:**
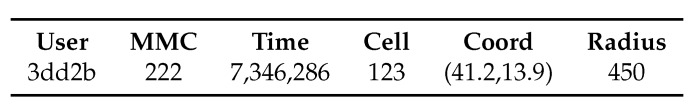
Structure of a call detail record (CDR). Every time a user sends or receives calls or text messages we generate one CDR with information about the user (hashed) id, the MMC (mobile country code), the timestamp of the CDR, and the code, coordinates and coverage radius of the cell tower.

In the context of this work, all this information serves as sporadic samples of the approximate locations of the phone’s owner. Specifically, the user’s location is given in terms of the cell network antenna the user was connected to. The area covered by a given antenna sector can be approximated by a circle with a given center and radius. Figure 1 shows the structure of a CDR. Each record comprises a user id (hashed), the MCC (Mobile Country Code) representing the country where the SIM card has been registered, the timestamp, the code of the cell tower along with its coordinates and coverage radius. Thus, the spatial resolution of CDR localization is the cell radius. Similarly to [40], in our work, we take into consideration different sectors for different antennas. Each sector is referred to as an individual cell and approximated with a circle (the circle-based representation of the cell’s coverage is provided directly from the telecom operator, and efficiently approximates the standard hexagonal model with sectored cells of 120-degrees [27,41]). It is worth noticing that, differently from a number of other works, we did not estimate the coverage of a cell network by using Voronoi tessellation. We stick to the computationally simpler representation of a cell as a circle with a given center and radius. In [42], it is shown that the approach does not change the accuracy of user location.

**Figure 2:**
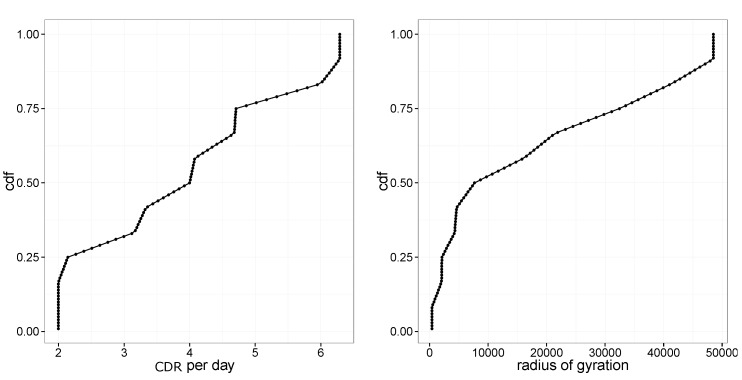
(**left**) Cumulative distribution (CDF) of CDRs per day. (**right**) CDF of radius of gyration.

Figure 2 illustrates some key statistics of our data. Figure 2-left illustrates the cumulative distribution (CDF) of CDRs per day. While the average number of CDRs per day is rather limited, we monitor a large user population comprising more than 4 million persons.

### 3.2. OD Matrix Estimation

Figure 2-right illustrates the CDF of a radius of gyration [43], illustrating the spatial extent of user traces, showing that almost half of the users are urban dwellers with $r_{g}$ less than 10 km. Users in the 50–75th percentiles are urban commuters as the diameter of peri-urban areas of main cities in the region is about 25–30 km. Users beyond the 75th percentile are associated with long-range commuters.

OD matrices are estimated in two steps. First, individual trips are identified, on the basis of either measured movements between cells (time-based trips) or of inferred routine trips known to be taking place (routine-based trips). Then, individual trips are aggregated to create the OD matrix.

Time-based trips. For each user we consider the sequence of his/her CDRs. For each pair $cdr_{i}$ and $cdr_{i+1}$, we assign a trip from the center of the $cdr_{i}$ cell to the center of the $cdr_{i+1}$ cell departing at time $t_{i}$ and arriving at time $t_{i+1}$ ($t_{i}$ and $t_{i+1}$ being the timestamps of the $cdr_{i}$, $cdr_{i+1}$ respectively).

Routine-based trips. We focus on home-work commute. We identify all the users generating CDRs in the area under study and apply the approach described in [44] to identify their home and work locations. This approach basically clusters the CDRs of each user into a set of areas. Then, it gives an importance weight to each of them on the basis of the user’s visiting pattern (e.g., areas typically frequented at night during the week will receive a high importance weight for the home place. An area typically frequented at working hours during the week will receive a high importance weight for the workplace). The weigh takes into consideration a number of behavioral characteristics. Finally, it selects those areas (i.e., clusters) best representing home and work locations with a dynamic threshold. The approach tolerates users with multiple homes or work locations, or even users without any.

Other than the location of home and workplaces, to compute commuting trips, it is fundamental to derive the time at which users travel. We developed a novel mechanism to estimate the time interval during which the user is in one of the home or work locations. Existing approaches [8] do not work well in our setting due to data sparsity. In fact, these approaches are based on averaging the departure/arrival time of trips starting/ending at home and work locations. In our setting, due to CDRs’ sparsity, it is difficult to obtain a large enough number of trips from home to work and vice-versa. Considering Figure 2-left, it is possible to see that 50% of the users have less than four CDR per day. Therefore the number of completed trips from home to work and vice-versa is very limited.

We compute instead, for each place, the temporal distribution of CDRs generated from that location, i.e., the distribution in terms of hours $h_{i}$ in which the user is present in that location. Our place identification approach [44] already clusters CDRs among multiple areas. Therefore, we can just consider all the CDRs in the cluster of the place under analysis (e.g., the cluster of CDRs associated with the user’s home). Given the set $h_{i}$ of hours in which the user is present in a location, we compute the associated distribution in terms of a circular mean μ and variance κ [45]. The use of circular statistics is of course needed to avoid that a person at home at 3 (3 a.m.) and 23 (11 p.m.) is considered at home at (3+23)/2=13 (1 p.m.). Circular mean and variance are defined as:

$$\mu =atan2(\sum_{i}^{n}sin\left(h_{i}\cdot \frac{\pi }{12}\right),\sum_{i}^{n}cos\left(h_{i}\cdot \frac{\pi }{12}\right))$$

$$\kappa =1-\frac{1}{n}\cdot \sqrt{(}{\left(\sum_{i}^{n}sin\left(h_{i}\cdot \frac{\pi }{12}\right)\right)}^{2}+{\left(\sum_{i}^{n}cos\left(h_{i}\cdot \frac{\pi }{12}\right)\right)}^{2})$$

Circular variance ranges from 0 (when all the CDRs happen at the same time) to 1 (when time difference between hours is 12—that is the max distance in the clock). Accordingly, κ·12 corresponds to the variation in hours associated with the data and thus μ±κ·12 roughly corresponds to the variability in our observations. Thus, for each place, we assume the arrival time at that place $t_{arrival}=\mu -\kappa \cdot 12$ and the departure time $t_{departure}=\mu +\kappa \cdot 12$.

On the basis of this information, for each user, we can create a simple, but user-specific trip-generation model that assumes that during each working day (Monday to Friday), each user undergoes a home–work commute with probability = 1 at a specific time. In the case of multiple homes or workplaces, the probability of a routine is equally spread across the alternatives (e.g., should the user have one home and two work places, she will commute to each work place with probability 0.5). These are rather simplistic assumptions that could be refined (via a prolonged analysis of people routine and behavior) in future work.

Data aggregation and OD matrices. Both the above approaches generate a set of trips taking place in a given day and time with a given probability (in the case of time-based trips the probability is always 1 as those are actually measured movements). OD matrices aggregate these trips together. Specifically, given a set of users, for a given day of the week and time interval Δt, we can build an OD matrix representing commuting trips on that day at that time, by taking into consideration either:

- All the trips starting within Δt. We refer to this as the starting time rule.
- All the trips ending within Δt. We refer to this as the ending time rule.

As we typically compute OD matrices on an hourly basis, a trip departing at 8:50 a.m. and arriving at 9:20 a.m. will be associated to the 8–9 a.m. (respectively, 9–10 a.m.) OD matrix in the case of the starting time rule (respectively, ending time rule). Assuming that the region under study is partitioned into a finite set of non-overlapping zones $S_{i}:i=1,..,n$, we can map each trip’s starting and ending location to the corresponding zones $S_{i}$ and $S_{j}$. In our current implementation, we have points representing the start and end locations of trips, therefore identifying the region *S* enclosing that point is straightforward. Advanced implementations can take into consideration the uncertainty in the localization of the start and end locations (e.g., the location might be a circle describing the coverage area of the network cell sector originating a CDR). In these cases, the probability of each trip will be partitioned among multiple zones proportionally to their overlap with start and end locations.

Calling $trip_{ij}$ the set of trips from $S_{i}$ to $S_{j}$, and $p\left(t\right),t\in trip_{ij}$ their probabilities, then $od_{ij}=\sum_{t\in trip_{ij}}p\left(t\right)$. Therefore, $od_{ij}$ counts the number of trips from $S_{i}$ to $S_{j}$. The resulting matrix describes—in a privacy conscious way as individual data is averaged out—the mobility patterns across the whole region.

### 3.3. Scaling

To match the actual number of users in the city, we scale the $od_{ij}$ counts to account for cell-phone usage and market penetration rates. As the ratio of cell phone users to the population is not uniform within the region, each user is assigned a home census municipality, and scaling factors are computed for each municipality by measuring the ratio of the assigned number of users and the reported population by national statistics. More specifically, considering home-to-work trips, $\forall i,\sum_{j}od_{ij}$ is the number of users living in $S_{i}$ and commuting from there. Similarly, considering work-to-home trips $\forall j,\sum_{i}od_{ij}$ is the number of users living in $S_{j}$ and going back home there. The actual number of users living in $S_{i}$ and with an occupation (therefore likely to undergo the home-work routine), named $C_{i}$, is available from census information, (http://www.istat.it/it/archivio/104317). On the basis of this information, we can scale home-to-work trips $od_{ij}$ by a factor of $C_{i}/\sum_{i}od_{ij}$. Similarly, we can scale work-to-home trips $od_{ij}$ by a factor of $C_{j}/\sum_{j}od_{ij}$. The result is an OD matrix actually matching the number of people living in the area.

### 3.4. Road Assignment

The above OD flows are already very useful in a number of applications to understand the overall mobility “demand” across the region. In addition, a further step is to map these flows to the road network, in order to understand traffic and congestions. On most city roads, free-flow speeds are rarely achieved due to congestion. As a result, traffic patterns may significantly change the time costs associated with routes. Following [7], we apply an incremental traffic assignment (ITA) algorithm that assigns batches of trips serially and updates traveling costs between increments based on the number of vehicles that were previously assigned to that road. Specifically, we divide each $od_{ij}$ flow into *K* batches each with a fraction $p_{k}$ of the total $od_{ij}$ flow. The origin/destination of each batch is assigned to a random starting/ending point within $S_{i}/S_{j}$. This distributes the flow in order not to create artificial congestion points and reflects general uncertainty in the exact starting and ending locations. Then we assign traffic to routes using the A* algorithm over the OpenStreetMap road network with the objective of minimizing travel time. Computations have been performed using the Graphopper library [46].

One of the most simplistic and common metrics used in determining the travel time associated with a specific flow level is the ratio between the number of cars actually using a road (volume) and its maximum flow capacity (volume-over-capacity or *V/C*). At low *V/C*, drivers enjoy large spaces between cars and can safely travel at free-flow speeds. As roads become congested and *V/C* increases, drivers are forced to slow down. Based on *V/C* for each road, costs are updated according to $t_{current}=t_{freeflow}\cdot \left(1+\alpha {\left(V/C\right)}^{\beta }\right)$ where α=0.15 and β=4 are used per guidelines set by the Bureau of Public Roads [7]. For each road, volume V is the number of previously assigned trips. C is directly obtained from OpenStreetMap meta-information via the Graphopper library.

The result is a map associating to each road segment the estimated number of people traveling in that segment at that time.

## 4. Experiments

In this Section we provide experiments to evaluate the results of all the above stages. As highlighted in [26], in order to understand the veracity of the obtained OD matrices, it is important to analyze OD estimation from multiple perspective and test multiple approaches to validate results and multiple data with which to compare. In this section, we perform preliminary experiments to understand the home–work commute that is at the basis of our hw-OD matrices. Then we focus on evaluating OD-flows, scaling and road assignment.

### 4.1. Home–Work Commute

Analysis of the home–work commute is based on two main steps: (i) accuracy in home and workplace identification and (ii) accuracy in the estimation of commuting time between home and work. With regard to home and workplace identification, detailed experiments and evaluations have been reported in [44]. Here we present a graphical representation of the estimated residential distribution in the areas under analysis in comparison with 2011 census-based estimates.

**Figure 3:**
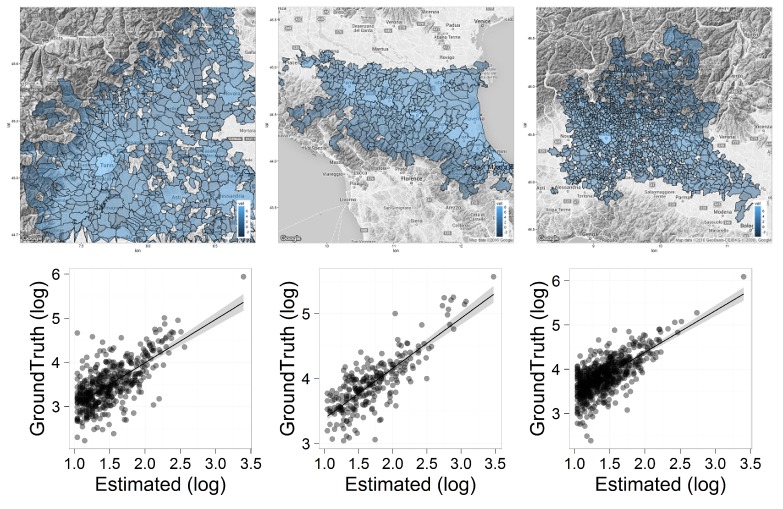
Density map for homes’ distribution and associated correlation with ISTAT Census in 2011. (**left**) Piemonte ($r^{2}=0.46$). (**middle**) Emilia Romagna ($r^{2}=0.64$). (**right**) Lombardia ($r^{2}=0.49$).

We used data from Piemonte spanning June 2015, from Emilia Romagna spanning April 2015, and Lombardia spanning March 2014 (choice dictated by data availability). In all the cases we considered only a sample of 20,000 users generating at least eight daily CDRs on average. For each user, we mapped his/her home location to the corresponding municipality (municipality areas are in the order of 10–50 km^2^). The result, shown in Figure 3, is a density map associating each area to the number of users (among the 20,000 mentioned above) with a home located in there. We compare such a density map with ground-truth information from ISTAT Census in 2011. Correlations are in Piemonte, $r^{2}=0.46$. In Emilia Romagna, $r^{2}=0.64$. In Lombardia, $r^{2}=0.49$.

We perform a correlation analysis between the two distributions, rather than measuring other kinds of errors (e.g., RMSE), because the census dataset has a much larger population. Therefore, as shown in the graph, the numbers involved are much larger. This would create extremely large errors, even if CDRs are able to correctly estimate where CDR users’ live.

The analysis of commuting time is more difficult as information about people commuting habits is intrinsically noisier and thus ground truth is harder to obtain.

**Figure 4:**
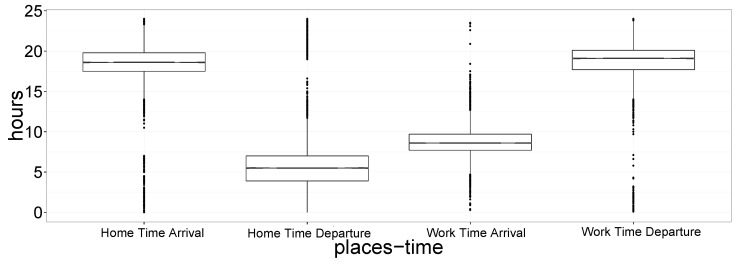
Distribution of all the estimated $t_{arrival}$ and $t_{departure}$ for home and work locations. Considering median time, people leave home at about 6 a.m. at arrive at work at 8 a.m. Then, they leave work at about 7 p.m. and arrive at home at the same time.

In Figure 4 we report a box plot of all the estimated $t_{arrival}$ and $t_{departure}$ for home and work locations. Considering the median time, it is possible to see that people leave home at about 6 a.m. and arrive at work at 8 a.m. Then, they leave work at about 7 p.m. and arrive at home at the same time. The box plot shows that data is highly concentrated and almost all users follow the same routine. These results conform to reasonable expectations.

One source of data to validate these results is the analysis on commuting for work and study purposes, collected in 2011 by the Italian National Institute of Statistics (http://www.istat.it/it/archivio/139381). This dataset records the origin-destination matrix of journeys for work or study reasons referred to as the resident population found at the 15th General Population Census (2011). The dataset contains the information on the number of people moving between municipalities—or within the same municipality—classified, in addition to the reason for the displacement, according to sex, means of transport used, departure time slot and duration of the journey. The basis of calculation is the 28,871,447 people who have declared to go to the usual place of study or work every day.

**Figure 5:**
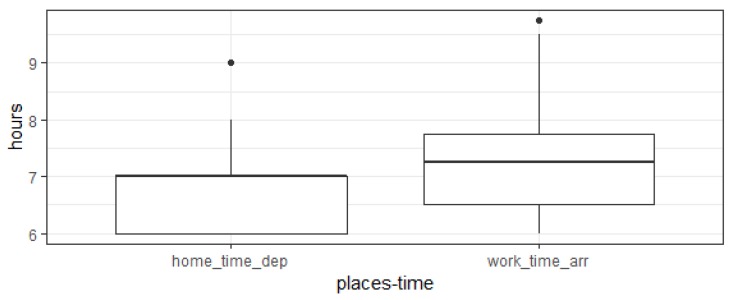
Distribution of home time departure and work time arrival for the ISTAT data.

Specifically, this data reports the home departure time (to go to work) and average travel duration, therefore it is possible to compute the distribution of home time departure and work time arrival. Figure 5 reports the distribution of such variables. It is possible to see that there is a fair overlap with the ones computed in our experimentation.

### 4.2. OD Flows

In this section we present experiments evaluating OD matrices on the basis of both time-based and routine-based trips. We present multiple experiments showing the resulting OD matrix for a given hourly interval and correlation results with ISTAT based information on commuting habits (same used for the home-work commute scenario in Section 4.1).

**Figure 6:**
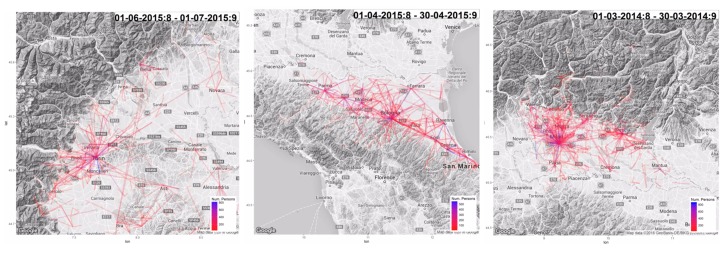
OD Matrices from home-work commute. Blue represent high flows, red low flows. (**left**) Piemonte. (**center**) Emilia Romagna. (**right**) Lombardia.

Figure 6 illustrates OD matrices derived from CDR data. Specifically, they are built on the basis of routine-based trips (computed over 1 month of data, for 20,000 users who generate at least 8 CDRs per day on average). Trips were scaled according to per-municipality market penetration. Lines represent the home–work commute taking place between 7 a.m. and 8 a.m. Supplementary videos for whole day analysis and results are available as Supplementary material (http://tiny.cc/41beaz).

**Figure 7:**
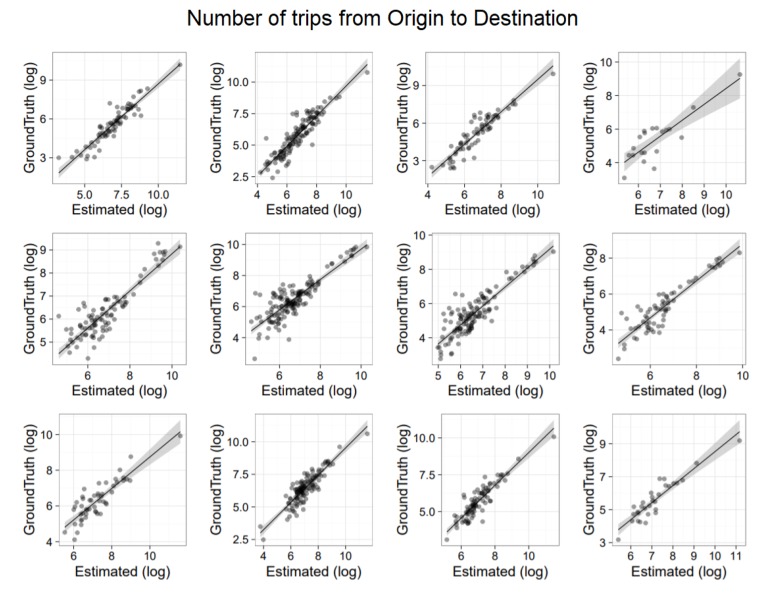
Comparison between the home–work commute with ISTAT data. (**top row**) provides results for Piemonte. (**middle row**), for Emilia Romagna. (**bottom row**), for Lombardia. In each row, we have four correlations associated to the four time intervals in which ISTAT data is organized, that is travel from home to work (**i**) before 7 a.m.; (**ii**) from 7 a.m. to 8 a.m.; (**iii**) from 8 a.m. to 9 a.m.; (**iv**) after 9 a.m.

**Figure 8:**
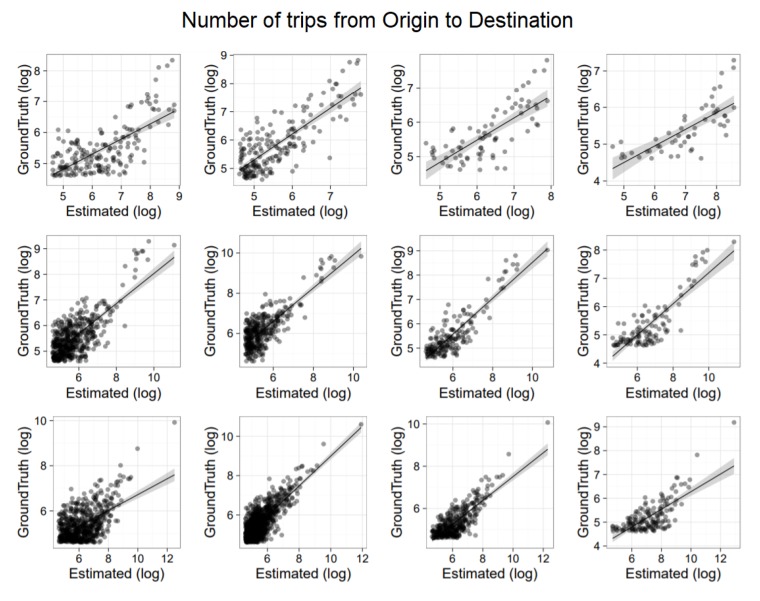
Comparison of time-based matrices with ISTAT. (**top row**) provides results for Piemonte. (**middle row**), for Emilia Romagna. (**bottom row**), for Lombardia. In each row, we have 4 correlations associated to the four time intervals in which ISTAT data is organized, that is travel from home to work (**i**) before 7 a.m.; (**ii**) from 7 a.m. to 8 a.m.; (**iii**) from 8 a.m. to 9 a.m.; (**iv**) after 9 a.m.

To evaluate the accuracy of the extracted OD matrices, we show a correlation analysis with ISTAT based information. Specifically, we compared ISTAT data at the municipality level with both routine-based and time-based matrices. Results are depicted in Figure 7 and Figure 8, respectively. It is worth noticing that while in the former analysis we use a generic day modeled via the commuting pattern, in the latter analysis we selected a specific working day. Moreover, we applied scaling (using per-municipality market penetration) only in the routine-based case (Figure 7) because only in that case home locations were available. In both figures, the top row provides results for Piemonte, the middle row for Emilia Romagna and the bottom row for Lombardia. Each row shows four correlations associated to the four time intervals in which ISTAT data is organized: travel from home to work (i) before 7 a.m.; (ii) from 7 a.m. to 8 a.m.; (iii) from 8 a.m. to 9 a.m.; (iv) after 9 a.m. All graphs are in log–log scale. On the *x*-axis there is the flow estimated with CDRs. On the *y*-axis there is the ISTAT based information.

Figure 7 (routine-based trips) presents correlation results with an average $r^{2}=0.8$. Figure 8 (time-based trips) presents correlation results with an average $r^{2}=0.5$. This is expected, as time-based trips capture all mobility aspects that might be peculiar of the selected day but also not related to home-work commute.

**Figure 9:**
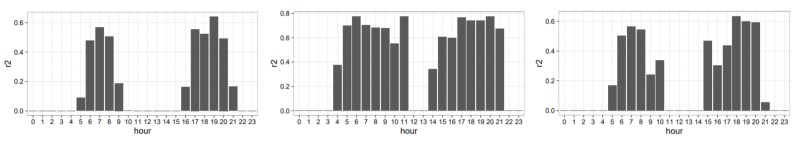
Comparison of time-based matrices with routine-based matrices. (**left**) Piemonte. (**middle**) Emilia Romagna. (**right**) Lombardia. For each hour we report the $r^{2}$ coefficient of the correlation between the two distributions.

In Figure 9, we also provide correlation analysis between routine-based and time-based matrices. As expected, stronger correlation takes place in early morning and later afternoon, where most of the traffic is associated to home-work commute. In central hours and at night, correlation drops almost to zero as very few home-work commute is expected to take place at that time.

### 4.3. Road Assignment

**Figure 10:**
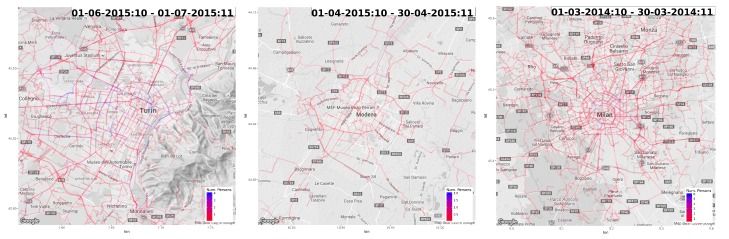
OD matrices from home-work commute mapped to the road network. Blue represent high flows, red low flows). (**left**) Torino. (**center**) Modena. (**right**) Milano.

In this set of experiments, we applied the iterative traffic assignment algorithm to project OD flows to the road network. We applied the approach to the matrices generated on the basis of home-work commute. In order to better emphasize the effect of road projection, we focus the analysis only in the city center. Specifically, we considered a 10 km^2^ area around each city center. We tessellate the area with a regular 10×10 grid. We ran OD matrix estimation in that partitioned area, and we applied iterative traffic assignment to project traffic from one cell to the other. Results are reported in Figure 10. Supplementary videos for whole-day analysis are available as supplementary material. (http://tiny.cc/41beaz).

**Figure 11:**
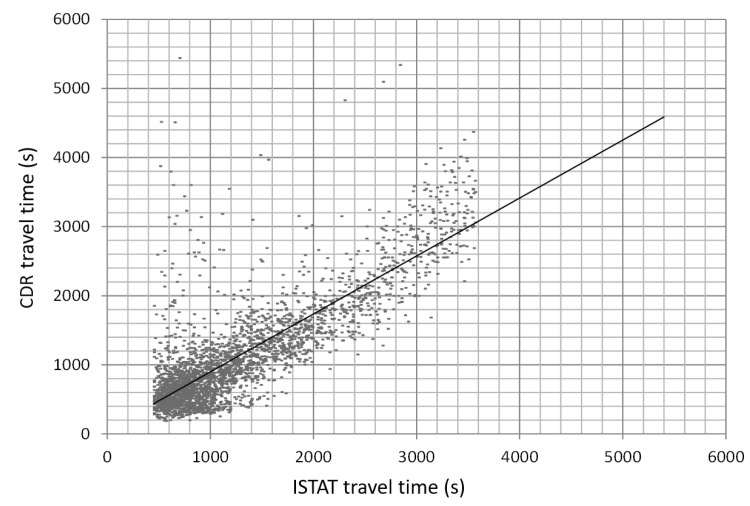
Comparison between travel times resulting from our approach and travel time from official statistics—www.istat.it/it/archivio/139381.

In order to validate our results, we compared travel time for home-work commuting resulting from our approach and from surveys conducted by ISTAT. (www.istat.it/it/archivio/139381). ISTAT data reports the home municipality, the work municipality and the commuting time for about 29,000 people. We aggregated data so as to obtain for each home–work municipalities the average commuting time. Similar estimates have been obtained from our approach, by averaging the travel time in HW-matrices. Correlation between the two estimates is reported in Figure 11. It is interesting to notice the set of points in which our results indicate a large travel time, while ISTAT reports a much shorter time. We tried to investigate some of these points (generating large errors). In several situations, they are associated with municipalities in the mountains. Our traffic assignment algorithms randomly assign home/work locations uniformly across the municipality area. Placing home–work locations on the “wrong” side of the mountain can notably bias results.

In a further set of experiments, we tried to validate our approach by comparing resulting road-level traffic as measured by our procedure and by Google Maps. Google Maps can provide both a real-time overlay of the current road-level traffic and the typical road-level traffic for that day of the week at a specific time. Since we are working with past data, we compared our results with the typical traffic at the corresponding time. As Google Maps APIs do not provide access to traffic data, we applied a screen scraping approach:

1. We perform a screen capture of Google Maps with the typical traffic for a given day of the week and time.
2. We plotted our results using Google Maps API in order to obtain a map similar to the official one, and we screen-captured also our results.
3. We applied a simple image alignment process, in order to align all the screen captures at pixel-level.
4. We thresholded the images pixel-by-pixel to remove the background and just leave the color-coded road-level traffic.
5. We computed a confusion matrix by pixel-by-pixel comparisons on the threshold images.

**Figure 12:**
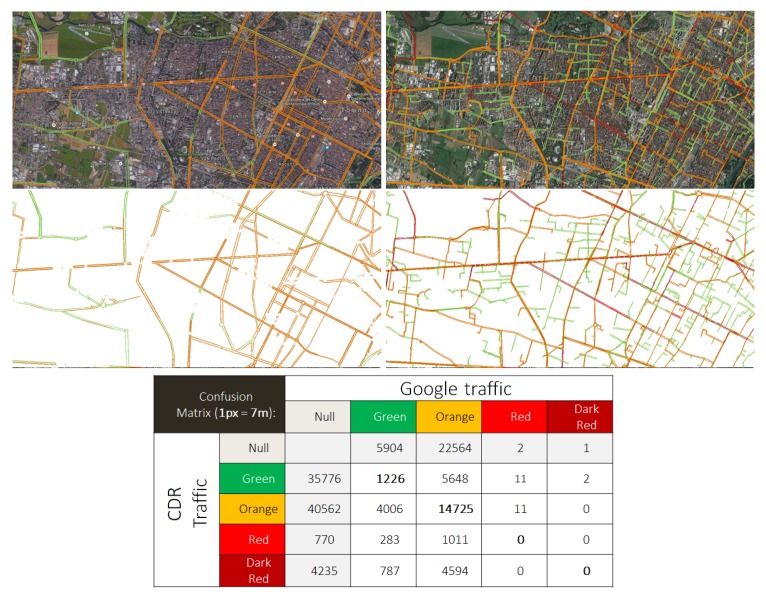
A comparison between Google and CDR-extracted traffic models for Torino. (**left**), Google traffic model—typical Tuesday—9 a.m.; (**right**), CDR traffic model—typical working day—9 a.m.; (**Below**), the related confusion matrix is reported.

Figure 12 shows an example of the screen capture and threshold procedure and the resulting confusion matrix. While this analysis is still preliminary, it is possible to see the alignment between the two maps. The main differences are due to pixels in which our approach assigns low traffic (green) while Google does not report any. We think that this is due mainly to how Google decides to display traffic information not to overload the user.

**Figure 13:**
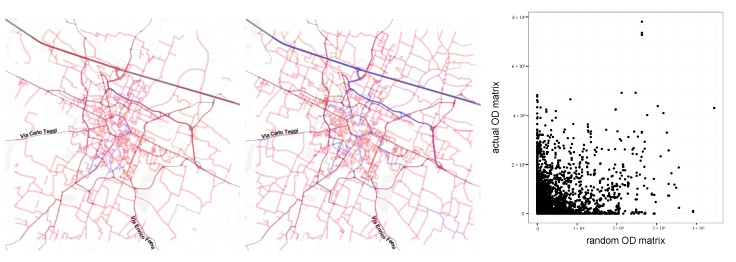
(**left**) Exemplary traffic result from original OD matrix. (**center**) Same traffic from random OD matrix. (**right**) Scatter of the road-level traffic counts resulting from the two OD matrices.

To better evaluate the extracted OD matrices, we run an experiment to compare traffic results obtained from the OD matrices and traffic results obtained from random OD matrices with the same number of trips. The idea is to understand how much information about road traffic is contained in the OD matrix and how much information is contained in the layout of the transportation network. More in detail, we create a random OD matrix by assigning to each o-d pair a number of trips obtained by sampling values from the original matrix with replacement. We applied to this matrix the same procedure described before: scaling and road assignment. We then analyze the correlation by the traffic in each road resulting from the original OD matrices and random ones. Figure 13 illustrates the results. In particular Figure 13-right shows the resulting correlation. In all the experiments $R^{2}<0.1$ indicating that most of the information is in the OD matrix and the layout of the transportation network seems to explain less than 10% of the resulting traffic.

## 5. Conclusions

In this work, we analyzed algorithms to compute time-based matrices (tOD) and routine-based matrices, in particular, associated with the home–work commute (hwOD) from CDR data, projecting those matrices onto the actual road network. We also described mechanisms to validate the resulting OD matrices by comparing it with several existing data sources. We compared results with census-based statistics obtaining correlations results: $r^{2}\approx 0.5$ for tOD and $r^{2}\approx 0.8$ for hwOD. In our future work, we intend to improve our analysis by performing comparisons with other information from the national statistics office, from other sensors (e.g., traffic cameras and sensors), and with standard mobility models such as gravity and radiation models [19]. We will also investigate the development of a model to integrate tOD and hwOD matrices. Further refinements could derive from the improvement of road traffic assignment to take into account individual travel preferences [37,47]. It could be also possible to perform operations on OD matrices, for example by subtracting the home-work OD matrix from time-based OD matrix, so as to highlight non-routine travels, and to build an automatic traffic bulletin system on that basis.

## Figures and Tables

**Table 1:** State of the art in OD matrices estimation from call detail records (CDR) and GPS data.

Ref.	OD Matrix	Scaling	Road Assignment	Evaluation
[5,6]	time-based	census	N/A	census correlation at district level (r=0.6), at city level (r=0.87)
[7]	time-based	census	incremental ($A^{*}$ with road weights depending on previous assignments)	census correlation (r=0.6)
[8]	home-work or other commute	census	incremental ($A^{*}$ with road weights depending on previous assignments)	census correlation at district level (r=0.55), at city level (r=0.98)
[25]	time-based	scaling OD to numbers from traffic cameras	incremental—traffic (micro)simulator	traffic camera correlation (RMSE = 335.09, RMSPE = 13.59%)
[28,29,30]	home-work commute	no	free-flow	no
[31]	time-based	N/A	A* with road weights depending on cells visited in the path	corresponding GPS traces (70 m median error)
[32]	time-based	census	training phase in which the handoff signature associated with a given road trip is computed and a (nearest neighbor) classifier is trained on that data	census at road level (r=0.77)
[33]	time-based	N/A	free flow	gravity model and region level (r=0.93)
